# Elucidating the interaction between light competition and herbivore feeding patterns using functional–structural plant modelling

**DOI:** 10.1093/aob/mcx212

**Published:** 2018-01-24

**Authors:** Jorad de Vries, Erik H Poelman, Niels Anten, Jochem B Evers

**Affiliations:** 1 Wageningen University, Laboratory of Entomology, Wageningen, The Netherlands; 2 Wageningen University, Centre for Crop System Analysis, Wageningen, The Netherlands

**Keywords:** *Brassica*, *nigra*, competition, functional–structural plant modelling, growth–defence trade-off, herbivore specialization, herbivory, plant–herbivore interactions, red far-red ratio

## Abstract

**Background and Aims:**

Plants usually compete with neighbouring plants for resources such as light as well as defend themselves against herbivorous insects. This requires investment of limiting resources, resulting in optimal resource distribution patterns and trade-offs between growth- and defence-related traits. A plant’s competitive success is determined by the spatial distribution of its resources in the canopy. The spatial distribution of herbivory in the canopy in turn differs between herbivore species as the level of herbivore specialization determines their response to the distribution of resources and defences in the canopy. Here, we investigated to what extent competition for light affects plant susceptibility to herbivores with different feeding preferences.

**Methods:**

To quantify interactions between herbivory and competition, we developed and evaluated a 3-D spatially explicit functional–structural plant model for *Brassica nigra* that mechanistically simulates competition in a dynamic light environment, and also explicitly models leaf area removal by herbivores with different feeding preferences. With this novel approach, we can quantitatively explore the extent to which herbivore feeding location and light competition interact in their effect on plant performance.

**Key Results:**

Our results indicate that there is indeed a strong interaction between levels of plant–plant competition and herbivore feeding preference. When plants did not compete, herbivory had relatively small effects irrespective of feeding preference. Conversely, when plants competed, herbivores with a preference for young leaves had a strong negative effect on the competitiveness and subsequent performance of the plant, whereas herbivores with a preference for old leaves did not.

**Conclusions:**

Our study predicts how plant susceptibility to herbivory depends on the composition of the herbivore community and the level of plant competition, and highlights the importance of considering the full range of dynamics in plant–plant–herbivore interactions.

## INTRODUCTION

Plants face a multitude of threats over the course of their development and have to balance resource allocation in response to these threats to maximize their fitness. Plants have to compete with neighbouring plants for resources such as light as well as defend against defoliation by herbivorous insects. Both these mechanisms require an investment of limiting resources such as nitrogen, and can drive selection towards an optimal distribution of these resources in the canopy (Stockhoff, 1994; Hikosaka *et al.*, 2016). Additionally, due to the potential costs of defences and the strong selective pressure of competition, plants are subjected to trade-offs in the allocation of limiting resources to either growth- or defence-related traits (Zust and Agrawal, 2017). These growth–defence trade-offs are strongly determined by spatiotemporal processes driven by interactions between plants and the plant and insect communities of which individual plants are a part (de Vries *et al.*, 2017). Plants have developed a highly variable ontogenetic trajectory in growth–defence allocation because plant tolerance to herbivory, the relative costs of defences as well as the selective pressure of competition fluctuate over the course of plant development (Barton and Boege, 2017). Resource-limiting conditions promote allocation of resources towards organs that are most favourably positioned relative to resource distributions (McKey, 1974). There has been a whole body of literature that has analysed the spatial allocation of resources in relation to maximizing canopy photosynthesis, growth and competitive ability (see reviews by, for example, Hirose, 2005; Hikosaka *et al.*, 2016). In dense vegetation, plants generally allocate most nutrients and especially nitrogen to leaves that are in the highest, most illuminated parts of the canopy and therefore contribute most to photosynthesis (Hirose *et al.*, 1987). This selective allocation pattern is believed to maximize whole-plant photosynthesis and is believed to be especially important in a competitive environment (Boonman *et al.*, 2006; Izaguirre *et al.*, 2013) where resource gradients are more pronounced, as exemplified by the increased light extinction in dense canopies. Leaves with a high light capture are vital to plant performance in a competitive environment due to their high contribution to plant carbon gain. This high light capture leads to selective investment of limiting resources to maximize the carbon gain from these leaves, further increasing their value. Models based on these principles are now widely used in basic plant ecological research (see Hirose, 2005), as well as in, for example, crop scientific and climate change studies (see Dewar *et al.*, 2009). However, an issue that these studies often overlook is the feedback that these resource allocation patterns in the canopy have on the distribution of herbivores and defences in the canopy.

The heterogeneity in nutritional value and defensive status of leaves also affects the spatial distribution of herbivores in the canopy, potentially threatening plants with the removal of leaves vital to their performance. Herbivore species differ in their susceptibility to taxon-specific secondary metabolites (Bennett and Wallsgrove, 1994) and can be roughly classified into two types: generalist and specialist species. Generalist species are not particularly resistant to any one secondary metabolite but can feed from a variety of host plants, avoiding those with high levels of secondary chemistry (Feeny, 1976). In contrast, specialist herbivores have adapted their metabolism to be more tolerant to taxon-specific secondary plant metabolites, but require a host of that particular taxon. Similarly, generalists are known to feed on less defended leaves lower in the canopy due to their lower resistance to plant defences, which are concentrated in young leaves in the top of the canopy (Cates, 1980). Conversely, a specialist’s increased resistance to plant defences allows them to target the young leaves that in dense vegetation are usually produced in the top of the canopy, which have a higher nutritional value but are also better defended. Therefore, generalist and specialist herbivores potentially have a different impact on the optimal allocation of defences and how growth–defence trade-offs affect plant fitness. However, it is unknown how the spatial distribution of herbivores interacts with competition-driven dynamics in resource gain and allocation, and how this impacts plant defence responses and growth–defence trade-offs.

Leaves at the top of the canopy that are preferred by specialist herbivores are vital to plant survival in a competitive environment, potentially aggravating the negative effect of specialist herbivores on plant fitness in such a competitive environment (de Vries *et al.*, 2017). Plants respond to impending competition with a set of morphological changes that maximize light capture in a competitive environment, including enhanced elongation of internodes and petioles, increased leaf angles and reduced branching. This set of responses is termed the shade avoidance syndrome (Fraser *et al.*, 2016; Ballaré and Pierik, 2017), and is mediated by the ratio of red to far-red light in the light spectrum (R:FR), among other signals. This light signal is a solid predictor of impending competition, as plant tissues absorb red light, while they reflect and transmit most of the far-red light (Ballare *et al.*, 1990; Ballaré and Pierik, 2017). A low R:FR indicates that light conditions are likely to be unfavourable in the near future and is known to affect plant defences negatively (Moreno *et al.*, 2009; de Wit *et al.*, 2013; Izaguirre *et al.*, 2013; Ballaré, 2014; Campos *et al.*, 2016). Competition creates heterogeneity in the light conditions and therefore creates heterogeneity in the distribution of defences in the canopy (Ballare *et al.*, 2012; Ballaré, 2014). This competition-induced heterogeneity in nutritional value and defensive status of leaves in turn affects the spatial distribution of herbivore damage depending on the herbivore species attacking the plant.

The interaction between plant–plant competition and herbivory can thus only be understood in a spatially dynamic context, which is not supported in conventional modelling tools, hampering quantitative analysis. To overcome this, we used the three-dimensional modelling techniques of functional–structural plant (FSP) modelling (Vos *et al.*, 2010) as a tool to assess how competition affects plant susceptibility to herbivores with different levels of specialization. FSP modelling is a promising tool to study plant–plant–herbivore interactions (de Vries *et al.*, 2017). The FSP model used in this study is novel through the incorporation of several interacting dynamic processes which are applied to the novel field of modelling plant–herbivore interactions. The model dynamically simulates plant–plant–herbivore interactions by implementing mechanistic source–sink-driven plant growth (Evers, 2016), R:FR-mediated architectural development (Bongers *et al.*, 2014) and the introduction of herbivores using agent-based modelling concepts. This allowed us to model both the direct effects of competition and herbivory on plant performance, and the indirect effects resulting from the interaction between herbivory and competition. First, we define our model and then address the question of how generalist and specialist herbivores affect plant fitness at varying levels of competition.

## MATERIALS AND METHODS

In this study we conducted two field experiments that serve to parameterize and validate the FSP model on plant growth and herbivory, and one greenhouse experiment to parameterize caterpillar feeding and growth. Model performance was evaluated by comparing simulations with experimental data, after which the model was used to elucidate the interaction effect of herbivory and plant competition for light.

### Experimental design

To parameterize and validate the model, we conducted two field experiments in 2014 and 2015 in Wageningen, the Netherlands. Plants of *Brassica nigra* were planted in three densities (1, 4 and 25 plants m^–2^) in plots of 2 × 2 m, with three replicates per density. Seeds were germinated in a greenhouse compartment with a 16 h/8 h light/dark photoperiod (20–22 ºC, 50–70 % relative humidity) and transferred to soil cubes as seedlings before being planted at 2 weeks old on 21 May (both years). Plots were weeded and sprayed with herbicides and pesticides weekly to exclude interspecific competition and herbivores. In 2014, three plants per plot (*n* = 9 plots per treatment) were randomly selected for weekly non-destructive measurements of plant height, total number of leaves along the main stem, the length and width of those leaves and the length of main stem internodes. Plants were selected from the entire plot (1 plant m^–2^), the inner 3 × 3 plants (4 plants m^–2^) or the inner 4 × 4 plants (25 plants m^–2^) to minimize border effects on measured values. Single measurements on leaf angles, branching angles, ear development, photosynthetic capacity and light quality (R:FR) were conducted at different moments during the experiment (Supplementary Data Methods S1). At the end of the experiment, we counted the number of branches per plant, measured internode thickness along the main stem and at branch bases, and harvested seeds to determine yield and average seed weight. Gathering seeds for yield measurements prevented us from sampling plants for destructive measurements during the first experiment, which is why we repeated the experiment in 2015. In the second year, we quantified plant biomass at 124 d after sowing, which was after the onset of flowering but before all leaves senesced (Supplementary Data Fig. S1). We also measured internode tissue density, which was done by measuring the internode length and radius to determine the internode volume, and weighing the internodes after a 16 h drying period in a 70 ºC oven.

Additionally, we conducted a greenhouse experiment to parameterize caterpillar feeding and growth based on the small cabbage white (*Pieris rapae*; Pieridae). This is a specialist on *Brassicaceae* and a caterpillar average in terms of size and its feeding damage compared with other insect herbivores on *B. nigra.* The caterpillars originated from the stock rearing of the Laboratory of Entomology, Wageningen University, where they are maintained on Brussel sprout plants (*B. oleracea* var. *gemmifera* L. ‘Cyrus’) in climatized rooms at 20–22 °C, 50–70% relative humidity and a 16 h/8 h light/dark photoperiod. We measured plant damage and subsequent growth of 30 individual caterpillars in their development from first instar to pupation while feeding on leaves of *B. nigra*, which were grown in the same conditions as the Brussel sprouts on which the caterpillars were reared. The caterpillars were kept in Petri dishes in a greenhouse compartment with a 16 h/8 h light/dark photoperiod and a 20 ºC:16 ºC temperature cycle. We placed a young leaf cut from a *B. nigra* plant grown in the same greenhouse in the Petri dishes, with a piece of wet cloth wrapped around the stem to maintain leaf quality. The leaves were photographed before being placed in the Petri dishes and after 2 d of feeding so that the initial and damaged leaf area could be determined using ImageJ (Supplementary Data Fig. S2). Until pupation, every 2 d the caterpillars were weighed and the leaves refreshed. Only the caterpillars that turned into a healthy pupa (determined by colour and weight) were used for data analysis. The measurements were used to determine herbivore feeding rate in sqaure metres of leaf damage per gram of caterpillar mass per growing degree day (gdd; to correct for the effect of temperature on feeding rate), the caterpillar growth in grams per square metre of consumed leaf area, the caterpillar weight at which pupation was initiated and the time to pupation (Table 2).

### Model design

The model used in this study was designed to simulate mechanistically plant–plant–herbivore interactions using a 3-dl FSP modelling approach (Vos *et al.*, 2010). The model was implemented in the platform GroImp v1.5 (Hemmerling *et al.*, 2008) and simulated individual plants growing at various densities in which plants compete with their neighbours for light. The distribution of light interception in the 3-D scene was simulated using a Monte-Carlo path tracer embedded in GroImp (Hemmerling *et al.*, 2008). The light environment was modelled using both randomly arranged diffuse light sources and direct light sources spread over the solar path that takes latitude and the simulated day of year into account (Evers *et al.*, 2010). Border effects in the light environment were eliminated by replicating a plot of simulated plants 25 times in the *x* and *y* directions for the light model calculations. The light conditions experienced by the 625 copies of each individual plant were then averaged, effectively eliminating border effects. Limitation of growth by below-ground resources such as water and nutrients was not considered. The model consisted of several components: plant architectural development; carbon assimilation; allocation and growth; shade avoidance; and herbivore behaviour and growth. An elaborate technical description of the implementation of these mechanisms can be found in the model design supplement (Supplementary Data Methods S2).

#### Plant architectural development

Following FSP modelling principles (Evers, 2016), the plant architecture was represented using a repetition of elementary units called phytomers, which consist of an internode, a leaf and an axillary meristem. Vegetative phytomers were sequentially produced at the top of the growth axis by the shoot apical meristem. After having produced a set number of vegetative phytomers, the plant began flowering and the meristem produced an inflorescence, after which no further vegetative phytomers were produced on the shoot. Axillary meristems could grow and develop similarly to the apical shoot meristem to form branches. Branch initiation and abortion were simulated using cues that are related to apical dominance, assimilate availability and light quality (Evers *et al.*, 2011). The outgrowth of axillary meristems is delayed by the dominance of the apical meristem as long as it remained vegetative or until the axillary meristem reached a given age. Additionally, a meristem required favourable growing conditions (surplus assimilates) to break and develop into a branch, which could be aborted during its development in the case of unfavourable conditions (low assimilates, low R:FR). Details on the mechanisms of branch initiation and abortion and their implementation can be found in Supplementary Data Methods S2. Branching also determined internode thickness as this was modelled using a pipe model, where the cross-sectional surface area of an internode is equal to the sum of the cross-sectional area of daughter internodes. This was relevant for model performance as internode thickness drove allocation of assimilates to the stem, which was a major sink that competed for assimilates with leaves and seeds.

#### Carbon assimilation, allocation and growth

The leaf area of each plant in the simulated plot determined light interception and subsequent carbon assimilation through photosynthesis, which then fed back to plant growth and leaf area expansion. Therefore, the effect of competition for light on plant growth was an essential, yet emergent, property of this FSP model, and depended on plant density and canopy structure. The amount of photosynthetically active radiation (PAR) absorbed by each individual leaf was calculated using reflectance and transmittance coefficients generally applicable to plant tissue (Jacquemoud and Baret, 1990). Leaf carbon assimilation was calculated from the amount of PAR absorbed by the leaf and its basic photosynthetic capacity, which itself was a function of the fraction of PAR absorbed (Niinemets and Anten, 2009; Hikosaka *et al.*, 2016). Assimilates were then stored in a central carbon pool from which maintenance respiration and assimilates needed for growth were deducted, and any remainder was kept as stored resources. Assimilates were distributed within the plant based on the relative sink strengths of organs, e.g. the assimilates needed to achieve potential growth (sink) relative to the sum of sink strengths of all growing organs in the plant (Heuvelink, 1996). This assumed no hierarchy for assimilate allocation between organs and took the added costs of growth respiration into account. The potential growth of an organ was described using the beta function for sigmoid growth (Yin *et al.*, 2003).

#### Shade avoidance

We used the light model to simulate the R:FR using reflectance and transmittance coefficients for red and far-red light generally applicable to plant tissue (Jacquemoud and Baret, 1990). This R:FR signal was perceived by the plant on the leaf tip and used to mediate both local and systemic integration of the shade avoidance response. Local mediation happens at the phytomer level, where the R:FR perceived by a leaf tip (Pantazopoulou *et al.*, 2017) mediated the shade avoidance response in that leaf as well as in the adjacent internode and lateral meristem. The R:FR signal mediated leaf width, leaf angle and internode length using a dose–response curve, while it mediated meristem outgrowth and abortion using a threshold method. A systemic integration of the combined R:FR signal of all main stem leaves regulated flowering time and potential leaf length, both using a threshold method of signal integration (Evers *et al.*, 2007).

#### Model calibration

We measured a number of parameters regarding plant architecture, development and growth that describe the plant phenotype in two different plant densities and serve as input to the FSP model (Table 1). These parameter values serve as a baseline description of *B. nigra* growth and development, based on the low-density phenotype. However, a number of growth parameters could not be measured directly in the field. To obtain values for these parameters, we used an optimization procedure where model output was fitted to measured data. Potential growth parameters of leaves and internodes as well as the effect of R:FR on these parameters were optimized such that the simulated biomass, yield, plant height and leaf length distribution patterns mimicked observed values. Note that organ size and, therefore, plant size and leaf area were thus model output rather than input, as they were the combined result of several growth parameters, mediation by R:FR, as well as carbon availability throughout plant development.

**Table 1.** Model parameters describing plant architectural development obtained through measurements or optimization

Parameter	Value	Unit	Acquisition
Leaf growth period	234	gdd	Measured
Leaf growth rate peak	167	gdd	Measured
Leaf length/width ratio	1.756 (2.5)*	Dimensionless	Measured
Leaf mass per unit area	65.8	g m^–2^	Measured
Internode growth period	258	gdd	Measured
Internode growth peak	216	gdd	Measured
Initial internode radius	0.0008	m	Measured
Internode density	176142	g m^–3^	Measured
Leaf appearance rate	12	gdd	Measured
Number of phytomers on the main stem	29 ± 5^†^ (27 ± 1)*	Dimensionless	Measured
Branch angle	40	Degrees	Measured
Leaf angle	70 (15)*	Degrees	Measured
Base photosynthetic capacity	30	µmol m^–2^ s^–1^	Measured
Potential leaf length	0.285 ± 0.026^†^	m	Optimized
Leaf length reduction by R:FR	0.55	Dimensionless	Optimized
Potential internode length	0.045 ± 0.01^†^	m	Optimized
Internode length increase by R:FR	3.5	Dimensionless	Optimized
Midpoint of the R:FR response	0.85	Dimensionless	Optimized
Steepness of the R:FR response	30	Dimensionless	Optimized
R:FR threshold for branch abortion	0.65	Dimensionless	Optimized

*A value in parentheses denotes that a separate parameter value is used for the high-density phenotype.

^†^Parameter drawn from a normal distribution with a mean and s.d., indicated with the mean ± s.d. notation.

#### Herbivore behaviour and growth

The simulated caterpillars were simulated as individual agents in the 3-D scene, and used random probability selection to choose a host-plant leaf upon instantiation and movement events. Every leaf was given a value that represented the relative probability (*rP*) that the leaf was selected by the individual herbivore. This value was described using a sigmoid curve based on temperature-corrected leaf age (*leafAge*), in gdd, the herbivore feeding preference (*h*, 0–1) and the parameters *rP*_max_ (maximum relative probability, denotes the difference between the youngest and oldest leaves), *s* (steepness parameter, determines the slope of the sigmoid curve for a given value of *h*) and *m* (midpoint of the sigmoid curve) (Supplementary Data Fig. S3).

$$rP=\frac{rP_{max}}{1+\text{exp}(s*\left(h-\left(1-h\right)\right)*\left(leafAge-m\right))}$$ (1)

Once the herbivore had chosen a host leaf, the herbivore feeding rate was described using a linear relationship with herbivore biomass (Table 2: Herbivore feeding rate). The herbivore then grew following a linear function based on the consumed leaf area (Table 2: Herbivore growth rate, *a* and *b*). When a leaf senesced, the herbivore moved to a new host leaf, had less biomass than the herbivore could consume during a time step or the herbivore developed into the next instar, which happened four times during its development at regular intervals. The herbivore pupated when it reached pupal age (Table 2: Herbivore life span), or when the herbivores mass reached a pupation threshold (Table 2: Maximum herbivore weight), effectively removing the herbivore from the 3-D scene. The simulated herbivore types differed in their feeding preference for older (*h* < 0.5) or younger leaves (*h* > 0.5), as is typical for the feeding behaviour of generalist and specialist herbivores, respectively.

**Table 2.** Model parameters describing herbivore feeding, growth and development based on *Pieris rapae*, a medium sized caterpillar commonly found on *Brassica nigra* plants

Parameter	Value	Unit
*a*_*w*_	10.61	g m^–2^
*b*	0.7747	Unitless
*a*_*f*_	7.68 × 10^–4^	m^2^ g^–1^ gdd^–1^
Initial herbivore weight	1.86 × 10^–5^	g
Maximum herbivore weight	0.178	g
Herbivore life span	210	gdd

Caterpillar weight gain (*c*_*w*_) is modelled using a two-parameter exponential function of caterpillar feeding rate (*c*_*f*_) (*c*_*w*_ = *a*_*w*_*c*_*f*_^*b*^), and caterpillar feeding rate is modelled using a linear function of caterpillar leaf area intake (*c*_*l*_) (*c*_*f*_ = *a*_*f*_*c*_*l*_).

### Model simulation design

#### Model evaluation

To evaluate model performance, we compared model output with measured data from the field experiments on the optimized variables leaf length, internode length and number of branches, as well as yield and biomass. The latter were validation variables of a higher integration level that resulted from the functional mechanisms of plant morphology, carbon allocation and shade avoidance. For example, plant biomass was largely determined by leaf area and branching patterns (Supplementary Data Fig. S4) which were variables at a lower level of integration. Complementary to the low and high density we also used an intermediate plant density (4 plants m^–2^) that was not used in model calibration to evaluate model performance. The simulations for model evaluation mimicked the set-up of the field experiments; we simulated three plant densities: low (1 plant m^–2^), intermediate (4 plants m^–2^) and high (25 plants m^–2^). The simulations were conducted with four, four and 16 plants per plot for the respective plant densities. These were replicated 625 times for the calculation of light interception distribution, resulting in plots of 2500, 2500 and 1000 simulated plants. Simulations ran from 31 March to 2 August (124 d), with average daily temperature, average daily insolation and solar angle typical for the Netherlands at a latitude of 52 °. The outputs gathered from these simulations were leaf area index (LAI) and R:FR (daily), leaf lengths, plant height, biomass and yield (after 124 time steps). Output from all plants in the plot was averaged and ten replicates were simulated.

#### Simulations

**Fig. 1.**
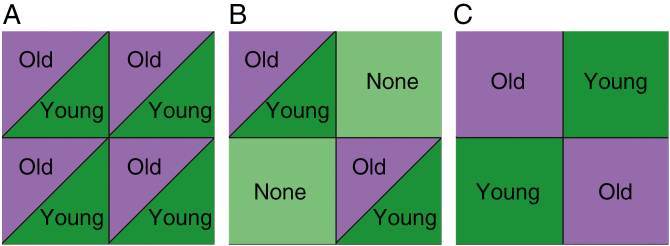
Three herbivore distribution patterns visualized with a block of four plants. (A) A homogeneous herbivore distribution where all plants are infested with the same herbivore type (i.e. herbivores preferring either old or young leaves). (B) A heterogeneous herbivore distribution following a checkerboard design of plants infested with the same herbivore type and non-infested plants. (C) An alternating herbivore distribution following a checkerboard design of plants infested with herbivores preferring young leaves and plants infested with herbivores preferring old leaves.

**Fig. 2.**
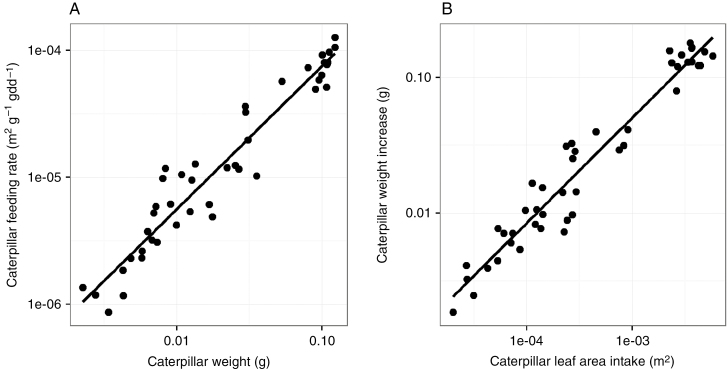
Correlation between measured data on caterpillar weight (g) and caterpillar feeding rate (m^2^ leaf area g caterpillar weight^–1^ gdd^–1^, *R*^2^ = 0.92) (A) and between caterpillar leaf area intake (m^2^) and subsequent caterpillar weight gain (g, *R*^2^ = 0.94) (B).

To investigate how herbivore feeding behaviour influences plant yield and interacts with plant competition for light, we conducted a full factorial set of simulations. These consisted of two plant densities (low, 1 plant m^–2^; high, 25 plants m^–2^), two herbivore types with different feeding preferences (preference for old leaves, *h* = 0.2; preference for young leaves, *h* = 0.8) and three herbivore distributions: (1) a homogeneous herbivore distribution where all plants were infested with the same herbivore type to get a baseline effect of herbivory on plant phenotype at a given plant density (Fig. 1A); (2) a heterogeneous herbivore distribution following a checkerboard design of plants infested with the same herbivore type and non-infested plants to test the interactive effect of herbivore damage and plant competition (Fig. 1B); and (3) an alternating herbivore distribution following a checkerboard design of plants infested with herbivores preferring young leaves and plants infested with herbivores preferring old leaves to test the effect of these two herbivore types on plant competitiveness (Fig. 1C). Plots contained four plants in the low density and 16 plants in the high density, and simulations ran from 31 March to 2 August (124 d), with average daily temperature, average daily insolation and solar angle typical for the Netherlands at a latitude of 52 °. Five, ten, 15 or 20 herbivores per plant were introduced after 60 d of growth (230 gdd), at this time the plants had accumulated enough leaf area to sustain the herbivores, and the plant phenotypes in low and high plant densities were still equal (Supplementary Data Fig. S5). The range in the number of simulated herbivores was chosen to represent the range of herbivores occurring in natural settings (Poelman *et al.*, 2009). We kept the number of herbivores per plant constant and equal for the two densities. Alternatives such as keeping the number of herbivores constant per unit ground area would result in either unrealistically high herbivore numbers on low-density plants (500 herbivores per plant) or irrelevantly low herbivore numbers on high-density plants (0.2 herbivores per plant). The output gathered from these simulations was cumulative herbivore damage, number of branches, leaf area, plant height, biomass and yield (after 124 d). The output of all plants in the plot was averaged and each treatment was replicated five times. The model output was tested for significance by conducting an analysis of variance (ANOVA) on the coefficients of a linear regression model. We tested for significance at the 5 % probability level. All average values are accompanied by a standard error, either through error bars in graphs or in textual form (mean ± s.e.).

## RESULTS

### Model calibration

A key feature of the model is simulation of the dynamic adjustment of the architectural phenotype in response to changes in plant density through changes in the light environment. Six variables that have a large impact on plant phenotype were observed to change with plant density in our experiments: leaf length distribution along the main stem, leaf angle, internode length, the number of phytomers in the main stem and the number of branches on the main stem. For our model, we assumed that these variables were all mediated by changes in R:FR as these responses fall within the framework of the shade avoidance syndrome (Ballaré and Pierik, 2017). The model was capable of simulating distinct differences in the R:FR over time between different plant densities (Supplementary Data Fig. S6). We optimized the simulated plant responses to a change in R:FR (Table 1) so that the phenotype of a simulated plant in high density represented the high-density phenotype observed in the field experiment.

### Herbivore feeding

Measured caterpillar weight (g) was strongly correlated with caterpillar feeding rate (m^2^ g^–1^ gdd^–1^, Fig. 2A). Subsequently, the amount of leaf area consumed by the caterpillar (m^2^) was strongly correlated with caterpillar weight gain (g, Fig. 2B). We parameterized the simulated caterpillars by fitting a function to the measured data; we used a linear function (*y* = *ax*, Table 2) to simulate caterpillar feeding rate as a function of caterpillar weight and a two-parameter power function (*y* = *ax*^*b*^, Table 2) to simulate caterpillar weight gain as a function of caterpillar leaf area intake.

### Evaluation of model performance

**Fig. 3.**
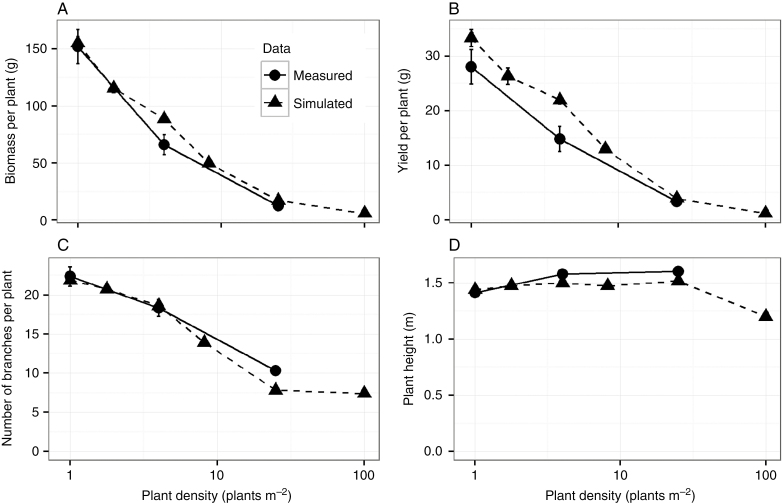
Comparison of measured and simulated data on plant biomass (A), plant yield (B), number of branches (C) and plant height (D) as a function of plant density. The measured data are collected at three plant densities whereas the simulated data cover six plant densities, showing the strength of model predictions beyond the parameterized densities (1 and 25 plants m^–2^).

Plant biomass and yield of seeds were used as proxies for plant performance and fitness, and were therefore our targets for evaluation of model performance. Measured plant biomass ranged from an average 152.2 ± 4.3 g d. wt at low density to 12.4 ± 0.2 g at high density (Fig. 3A) and measured plant yield showed a similar pattern, ranging from 28 ± 3.2 g dry seed weight at the low density to 3.3 ± 0.52 g at the high density (Fig. 3B). Simulated plants showed comparable correlation between plant density and both plant biomass and yield, with a slight overestimation of biomass and yield at an intermediate density.

The number of branches on the main stem was one of the main determinants of simulated plant biomass (*R*^2^ = 0.72, Supplementary Data Fig. S4) and yield of seeds (*R*^2^ = 0.70, Supplementary Data Fig. S4). The measured number of branches decreased with plant density, from 22.4 ± 1.2 at low density to 10.6 ± 0.5 at high density (Fig. 3C). Our FSP model was able to mimic the observed non-linear decrease of the number of branches with plant density by mediating the abortion of developing branches by the drop in simulated R:FR associated with increasing plant density (Supplementary Data Fig. S6).

Measured plant height ranged from 1.41 ± 0.04 m in the low plant density to 1.6 ± 0.04 m in the high density. The simulated plants had a height of 1.48 m at low density to 1.28 m at high density (Fig. 3D). Model underestimation of plant height at higher densities was potentially due to a border effect that was present in the measured plants but was absent in the model simulations. However, plant height contributed relatively little to the accumulation of biomass (*R*^2^ = 0.066, Supplementary Data Fig. S4) and yield (*R*^2^ = 0.12, Supplementary Data Fig. S4) and was therefore given less weight when optimizing model performance.

**Fig. 4.**
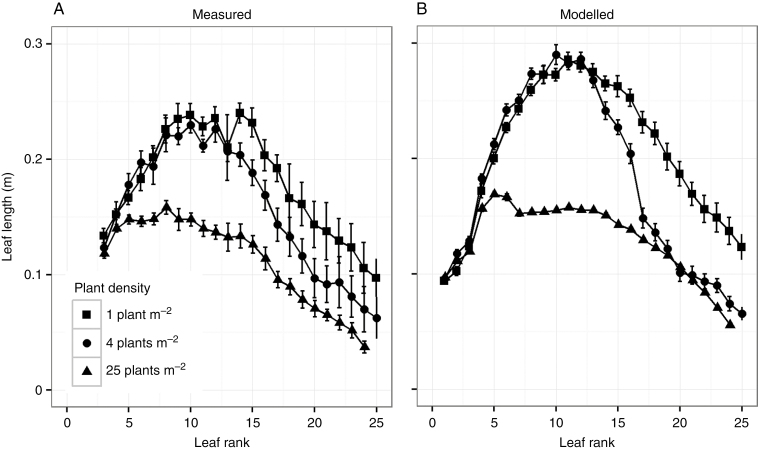
Leaf length per leaf rank on the main stem of *Brassica nigra* grown in three densities in the field (A) and simulated with the model (B). The high (25 plants m^–2^) and low densities (1 plant m^–2^) have been used for model calibration, while the middle density (4 plants m^–2^) served as validation. Up to leaf rank 25 is shown as not all plants produce enough phytomers to have leaves of higher ranks along the main stem.

Leaf area was another main determinant of simulated plant biomass (*R*^2^ = 0.94, Supplementary Data Fig. S4) and seed yield (*R*^2^ = 0.92, Supplementary Data Fig. S4) in the model. In field-grown plants, we observed differences in leaf length distributions along the main stem between densities; plants grown at high density had shorter leaves and a different length distribution along the main stem compared with plants grown in low density (Fig. 4). The leaf rank at which the leaf length values of plants from different densities deviated from each other gives a relative indication of the stage in plant development at which canopy closure initiated shade avoidance (Supplementary Data Fig. S6 and S7). Although leaf lengths were slightly overestimated by the model, the simulated pattern in the effect of plant density on leaf length distributions closely matched field data. Accurate depiction of this density effect on leaf length distributions is instrumental to evaluate the interaction between herbivory and plant density.

**Fig. 5.**
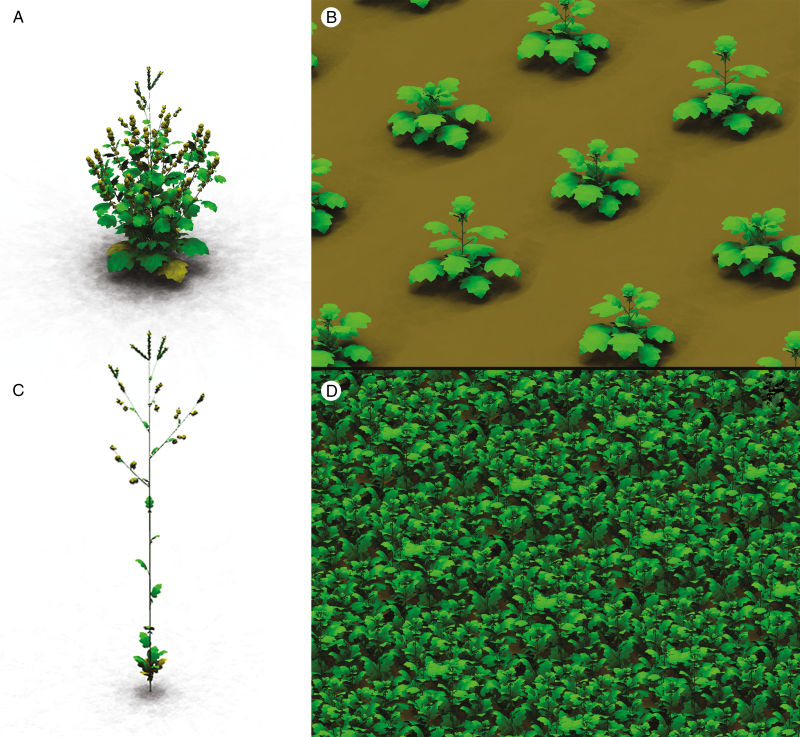
Simulated plant phenotype in a low density of 1 plant m^–2^ (A, B) and a high density of 25 plants m^–2^ (C, D), showing isolated plants in the generative stage (A, C) and a field of simulated plants in the vegetative stage (B, D).

In conclusion, the effect of plant density on the simulated plants was comparable with the effect of plant density on real plants. Both the simulated yields, which we used as a proxy for plant fitness, and the simulated variables that underlie the accumulation of yield (leaf length, branching patterns and biomass, Supplementary Data Fig. S4) showed patterns comparable with real plants. Therefore, we conclude that the model satisfactorily simulated the most important mechanisms that determined changes in plant phenotype in response to changes in plant density (Fig. 5).

### The effect of herbivory on plant performance

Using the model, we tested how herbivore feeding behaviour influenced plant yield and interacts with plant competition for light by simulating different levels of herbivory in two plant densities, two herbivore feeding preferences and three herbivore distribution patterns.

#### Density effects

**Fig. 6.**
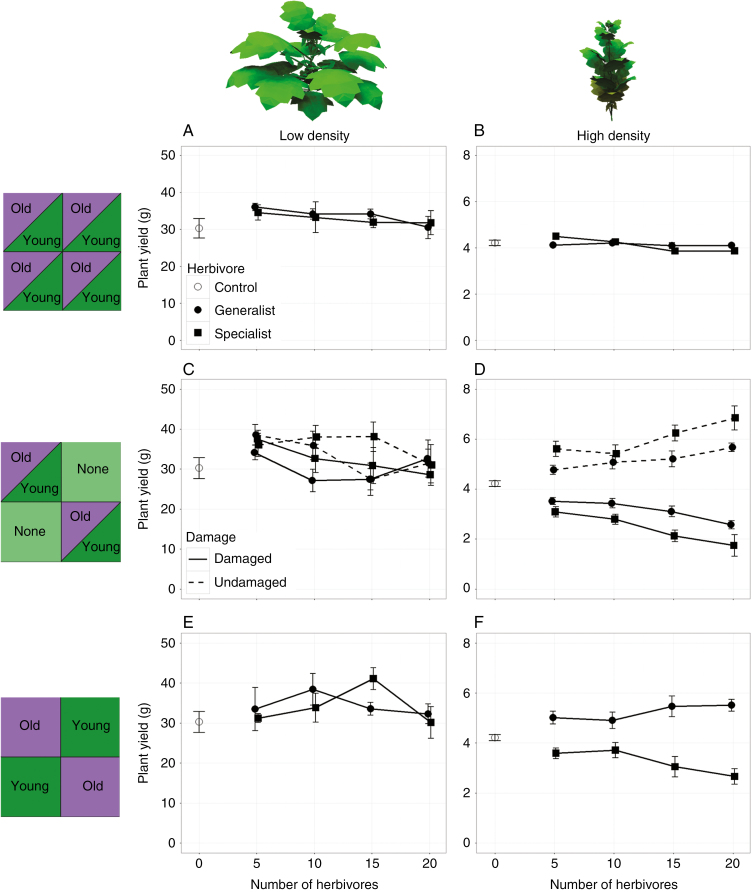
Simulated plant yield at day 124 plotted against the number of herbivores per plant in two densities (low, 1 plant m^–2^; high, 25 plants m^–2^) and three herbivore distribution patterns: homogeneous distribution of herbivores preferring either old or young leaves (A and B), a checkered pattern of undamaged plants (none) and plants infested by herbivores preferring old or young leaves (C and D) and a checkered pattern of plants infested by herbivores preferring old or young leaves (E and F). *n* = 5 in all cases.

From our simulations, a clear interaction effect between herbivory and plant density emerged (Fig. 6). Herbivory had no significant effect on simulated plant yield in any of the low-density treatments (Fig. 6A, C, E), whereas herbivory had a significant negative effect on simulated plant yield in all high-density treatments (Fig. 6B, D, F). This interaction between herbivory and density can be explained by three factors. First, low-density plants had considerably more leaf area (Supplementary Data Fig. S5), branches and biomass (Fig. 3A, C) than the high-density plants. These size differences decreased the relative impact of a single herbivore on low-density plants, which allowed the low-density plants to compensate damage more readily than the high-density plants. Secondly, the number of herbivores per plant was kept constant, resulting in more herbivores per unit ground area and plant biomass at the high density. Thirdly, the increased competition among plants in high densities magnified small herbivore-induced differences in plant biomass between neighbouring plants because of the resulting asymmetry in competitive strength. Additionally, what happened to the light interception lost by leaves because of herbivore damage should be considered. In low densities, some of that light was not intercepted by the plants at all, but most was intercepted by other leaves of the same plant instead. In the high plant density, however, part of that light was intercepted by other leaves of the same plant, whereas another part of that light was intercepted by leaves of other plants. This spillage of light to neighbouring plants gave them a competitive advantage over the damaged plant and caused asymmetry in the competition between these plants.

#### Treatment effects

The number of herbivores and the herbivore feeding preference had no effect on plant yield in a low plant density, regardless of the distribution of the herbivores. In a homogeneous distribution of herbivores in a high plant density (Fig. 6B), the number of herbivores had a small negative effect on plant yield (*F* = 14.97, *P* < 0.01) and herbivore feeding preference had a small effect (*F* = 6.41, *P* < 0.05). Additionally, herbivore density and feeding preference interacted (*F* = 7.02, *P* < 0.01), where herbivores preferring old leaves suppressed yield more than herbivores preferring young leaves at low herbivore abundance, and vice versa at high herbivore abundance. Although all these effects are highly significant, the relevance of these effects can be disputed as their magnitude was not very large. When infested plants were competing with uninfested neighbours (Fig. 6D), the number of herbivores suppressed the yield of infested plants (*F* = 28.11, *P* < 0.001). The herbivore feeding preference also affected the yield of infested plants (*F* = 19.05, *P* < 0.001), where herbivores preferring young leaves supressed yield more than herbivores preferring old leaves. However, the number of herbivores and herbivore feeding preference did not interact. Having both infested and uninfested plants within a plot created a competitive disadvantage for infested plants and a competitive advantage for uninfested plants, because the uninfested plants had more leaf area than the infested plants. This variation in leaf area and, consequently, in competitive strength was magnified by competition and translated into a yield difference that increased with the number of herbivores per plant. Additionally, this effect was greater for plants infested with herbivores preferring young leaves at the top of the canopy compared with herbivores preferring old leaves in the bottom of the canopy [*h*, Eqn (1)]. With a checkered distribution of plants infested with herbivores preferring young leaves and herbivores preferring old leaves (Fig. 6F), the herbivore feeding preference affected plant yield (*F* = 80.48, *P* < 0.001) and interacted with the number of herbivores (*F* = 7.79, *P* < 0.01). The herbivores preferring old leaves had a positive effect on plant yield that increased with the number of herbivores, whereas the herbivores preferring young leaves had a negative effect on plant yield that increased with the number of herbivores. The herbivores feeding on young leaves in the top of the canopy caused their host plants to have a competitive disadvantage compared with the plants infested with herbivores that fed on older leaves in the bottom of the canopy [*h*, Eqn (1)].

## DISCUSSION

Our results indicate that the costs of herbivory for plants may strongly depend on the type of herbivore attacking the plant and the extent of plant–plant competition under which plants are attacked by herbivores. Our model predicts that damage by either herbivores feeding on younger leaves or herbivores feeding on older leaves can be tolerated by plants growing in a low density. In a high plant density, the costs of herbivore attack are shaped by the interaction of herbivore feeding location and distribution of herbivory among neighbouring plants. Damage to both young and old leaves can be tolerated when neighbouring plants are equally damaged in both feeding location and density of the herbivores. However, damage to young leaves is more costly to the plant than damage to old leaves when neighbouring plants are less affected by herbivore damage, which causes a competitive advantage and a subsequent fitness benefit for the neighbouring plants. These conclusions show the need to link research on herbivory to research on plant–plant interactions, as the fitness costs and benefits of herbivory and defence are tightly linked to the plant’s competitive context.

Many experimental studies have reported that tissue loss from above-ground herbivores affects plant–plant competition by decreasing the competitive ability of one of the competitors (Rees and Brown, 1992; Hambäck and Beckerman, 2003; Haag *et al.*, 2004; Schadler *et al.*, 2007). Other studies have shown that preferential feeding of the herbivore impacted plant–plant interactions by increasing the competitive asymmetry between competing plants (Bentley and Whittaker, 1979; Kim *et al.*, 2013; Borgström *et al.*, 2016). These findings are in concordance with our results that show how a heterogeneous distribution of herbivores between plants impacts plant fitness by affecting the outcome of plant–plant competition. However, above-ground feeding by a generalist herbivore has been shown to affect plant performance without affecting the outcome of plant–plant competition (Jing *et al.*, 2015). These finding are in line with our model predictions as herbivores with a preference for older leaves can be seen as analogous to the feeding behaviour of a generalist herbivore. Future experimental work will have to validate our model predictions further and explore their implications in a complex ecological setting.

Young leaves at the top of the canopy represent current resource investment and future resource capture, and are, therefore, of high value to the plant. An increase in plant density reduces light capture of older leaves located in lower strata of the canopy and thereby increases the plant’s dependency on young leaves for future resource capture. Removal of these disproportionately valuable leaves by a herbivore robs the plant of multiple important resources (e.g. light and nitrogen), which potentially creates a strong competitive disadvantage relative to the neighbouring plants if those are not or are less severely attacked. Even a small competitive disadvantage can lead to a fitness loss for the damaged plant through the asymmetry of plant competition. Our results show both the disproportionately high value of young leaves under severe competition and the enhanced negative effect through the asymmetry of light competition when losing these leaves (Fig. 6). However, current models that relate resource allocation among leave to canopy photosynthesis, growth and competition (e.g. see review by Hirose, 2005) generally do not consider the risks and impact of herbivory. Additionally, we can consider how digestion of older vs. younger leaves has potential consequences for nutrient competition among plants through changes in litter composition. Secondary metabolites have been shown to serve alternative ecosystem functions, such as the role of tannins in nutrient cycling and retention through their effect on litter decomposition rates (Hättenschwiler and Vitousek, 2000). Therefore we advocate a stronger quantitative integration of herbivory and defence into existing resource allocation models, models of plant–plant competition and nutrient flux models.

Functional–structural plant models have been suggested as a useful tool to model dynamic interactions between plants (Vos *et al.*, 2010; Bongers *et al.*, 2014; de Vries *et al.*, 2017). However, most studies to date utilize a limited range of the possibilities provided by FSP modelling: some studies used descriptive FSP models to test static architectural traits (Chen *et al.*, 2014; Zhu *et al.*, 2015) or a dynamic response to an environmental variable of one architectural trait such as tillering to R:FR (Evers *et al.*, 2007). Other studies included dynamic growth rules based on carbon acquisition and allocation following source–sink principles, but do not include responses to environmental or neighbour-induced signals (Evers *et al.*, 2010; Lopez *et al.*, 2010; Evers and Bastiaans, 2016) or simulate plants in a static context (Kang *et al.*, 2012). For the present study, we developed a fully dynamic FSP model that simulated multiple plants competing for light, both through carbon acquisition and source–sink principles and through responses to a dynamically changing R:FR signal. In this way we captured the key mechanisms underlying growth and development of *Brassica nigra* under intraspecific above-ground competition, which allowed us to elucidate how leaf removal by an insect herbivore impacts plant fitness through the changes in light climate and the interaction with dynamic plant responses to neighbours. Plants respond to damage by insect herbivores by eliciting the production of secondary metabolites that serve as a defensive mechanism to deter or hamper the growth of the attacking herbivore. Our current model does not incorporate this defensive mechanism as parameterizations on both the plant and herbivores were done using non-elicited plants. Plant defence elicitation has a potentially large effect on model predictions and is a logical next step in the development of our plant–plant–herbivore FSP model.

Plant competition for light and defence against herbivory are known to interact through the same signal that induces shade avoidance growth, R:FR, which also downregulates plant defences (Moreno *et al.*, 2009; de Wit *et al.*, 2013; Izaguirre *et al.*, 2013; Ballaré, 2014; Campos *et al.*, 2016; Cortes *et al.*, 2016). This mechanism potentially allows the plant to optimize the allocation of its defences by prioritizing leaves that are of high value to the plant (Izaguirre *et al.*, 2013; de Vries *et al.*, 2017). Plants are known to elicit a stronger defence response in younger leaves (Koricheva and Barton, 2012), which is in line with our model predictions on the disproportionately high contribution of these leaves to plant competitiveness. This increase in secondary metabolism in young leaves is regulated by phytohormones such as cytokinins that also positively regulate primary metabolism (Giron *et al.*, 2013; Brütting *et al.*, 2017), hinting at a link between optimal resource and optimal defence allocation. When considering optimal defence allocation, we should take into account that defence allocation patterns change over the course of plant development and that these whole-plant defence trajectories are much more variable than can be expected from the ontogenetic defence trajectory at the leaf level (Barton and Boege, 2017). This is in part due to changes in the need to prioritize growth, reproduction, competitive strength or defence over the plant’s development (Boege and Marquis, 2005). The optimal balance between growth- and defence-related traits depends on the current plant phenotype and on external selective forces such as herbivory and light competition. Future elucidation of whole-plant phytohormonal regulation of both primary and secondary metabolism is needed to understand fully the functioning of growth–defence trade-offs and put the predictions of the FSP modelling into perspective. Additionally, future developments of the FSP model to include regulation of primary and secondary metabolism can help elucidate the adaptive value of these regulatory mechanisms and how ontogenetic trajectories in optimal defence allocation affect growth–defence trade-offs.

Specialist and generalist herbivores differ not only in their feeding preferences (Cates, 1980), but also in their colonization of a host plant. Specialist herbivores are known to oviposit preferentially on defended plants as they use plant defence chemicals as a host detection cue, whereas generalists often forgo these high defence phenotypes (Poelman *et al.*, 2008; Stam *et al.*, 2014). The feedback between plant defences and the herbivore community colonizing the plant can be an important driver of plant defence allocation (Poelman and Kessler, 2016). The herbivore community experienced by a plant is the result of complex interaction between the behaviour and abundance of different herbivore species, as well as the phenotype and ecological context of the plant. Our results suggest that the way a given herbivore community impacts plant fitness depends on the composition of that herbivore community as well as the level of competition the plant is facing. These results emphasize the importance of considering the full range of dynamics in plant–plant–herbivore interactions when looking at growth–defence trade-offs (de Vries *et al.*, 2017). A future challenge lies in elucidating the interaction of plant competition and herbivore community dynamics and how this interaction affects plant fitness. This challenge can be met by expanding our FSP model with plant defences and linking the plant defence phenotype to herbivore behavioural patterns on oviposition and feeding. These model developments add a level of dynamics to this already highly dynamic modelling approach and allow us to elucidate further the effect of dynamic plant–plant–herbivore interactions on plant fitness.

## SUPPLEMENTARY DATA

Supplementary data are available online at https://academic.oup.com/aob and consist of the following. Methods S1: supplementary experiments. Methods S2: supplementary model design. Figure S1: architecture of field-grown plants in low and high density. Figure S2: leaf damage by a caterpillar in the greenhouse experiment. Figure S3: simulated herbivore specialization and subsequent feeding preference. Figure S4: correlations between several simulated variables. Figure S5: simulated leaf area over time. Figure S6: simulated R:FR ratio over time. Figure S7: simulated leaf area index (LAI) over time.

mcx212_suppl_Supplementary_Information

## References

[CIT0001] Ballaré CL 2014 Light regulation of plant defense. Annual Review of Plant Biology 65: 335–363. 10.1146/annurev-arplant-050213-040145 24471835

[CIT0002] Ballaré CL, Pierik R 2017 The shade-avoidance syndrome: multiple signals and ecological consequences. Plant, Cell and Environment 40: 2530–2543. 10.1111/pce.12914 28102548

[CIT0003] Ballare CL, Scopel AL, Sanchez RA 1990 Far-red radiation reflected from adjacent leaves: an early signal of competition in plant canopies. Science 247: 329–332. 17735851 10.1126/science.247.4940.329

[CIT0004] Ballare CL, Mazza CA, Austin AT, Pierik R 2012 Canopy light and plant health. Plant Physiology 160: 145–55. 22802612 10.1104/pp.112.200733 PMC3440192

[CIT0005] Barton KE, Boege K 2017 Future directions in the ontogeny of plant defence: understanding the evolutionary causes and consequences. Ecology Letters 20: 403–411. 28145095 10.1111/ele.12744

[CIT0006] Bennett RN, Wallsgrove RM 1994 Secondary metabolites in plant defense mechanisms. New Phytologist 127: 617–633. 10.1111/j.1469-8137.1994.tb02968.x 33874382

[CIT0007] Bentley S, Whittaker JB 1979 Effects of grazing by a chrysomelid beetle, *Gastrophysa viridula*, on competition between *Rumex obtusifolius* and *Rumex crispus*. Journal of Ecology 67: 79–90.

[CIT0008] Boege K, Marquis RJ 2005 Facing herbivory as you grow up: the ontogeny of resistance in plants. Trends in Ecology and Evolution 20: 441–448. 16701415 10.1016/j.tree.2005.05.001

[CIT0009] Bongers FJ, Evers JB, Anten NPR, Pierik R 2014 From shade avoidance responses to plant performance at vegetation level: using virtual plant modelling as a tool. New Phytologist 204: 268–272. 25236169 10.1111/nph.13041

[CIT0010] Boonman A, Anten NP, Dueck TA et al 2006 Functional significance of shade-induced leaf senescence in dense canopies: an experimental test using transgenic tobacco. American Naturalist 168: 597–607. 10.1086/508633 17080359

[CIT0011] Borgström P, Strengbom J, Viketoft M, Bommarco R 2016 Aboveground insect herbivory increases plant competitive asymmetry, while belowground herbivory mitigates the effect. PeerJ 4: e1867. 27069805 10.7717/peerj.1867 PMC4824911

[CIT0012] Brütting C, Schäfer M, Vanková R, Gase K, Baldwin IT, Meldau S 2017 Changes in cytokinins are sufficient to alter developmental patterns of defense metabolites in *Nicotiana attenuata*. The Plant Journal, 89: 15–30. 27557345 10.1111/tpj.13316 PMC5245775

[CIT0013] Campos ML, Yoshida Y, Major IT et al 2016 Rewiring of jasmonate and phytochrome B signalling uncouples plant growth–defense tradeoffs. Nature Communications 7: 12570. doi: 10.1038/ncomms12570. 10.1038/ncomms12570 PMC5155487 27573094

[CIT0014] Cates RG 1980 Feeding patterns of monophagous, oligophagous, and polyphagous insect herbivores: the effect of resource abundance and plant chemistry. Oecologia 46: 22–31. 28310621 10.1007/BF00346961

[CIT0015] Chen T-W, Henke M, De Visser PH et al 2014 What is the most prominent factor limiting photosynthesis in different layers of a greenhouse cucumber canopy?Annals of Botany 114: 677–688. 24907313 10.1093/aob/mcu100 PMC4217677

[CIT0016] Cortes LE, Weldegergis BT, Boccalandro HE, Dicke M, Ballare CL 2016 Trading direct for indirect defense? Phytochrome B inactivation in tomato attenuates direct anti-herbivore defenses whilst enhancing volatile-mediated attraction of predators. New Phytologist 212: 1057–1071. 27689843 10.1111/nph.14210

[CIT0017] Dewar RC, Franklin O, Mäkelä A, McMurtrie RE, Valentine HT 2009 Optimal function explains forest responses to global change. BioScience 59: 127–139.

[CIT0018] Evers JB 2016 Simulating crop growth and development using functional–structural plant modeling. In: Hikosaka K, Niinemets Ü, Anten PRN, eds. Canopy photosynthesis: from basics to applications. Dordrecht: Springer Netherlands, 219–236.

[CIT0019] Evers JB, Bastiaans L 2016 Quantifying the effect of crop spatial arrangement on weed suppression using functional–structural plant modelling. Journal of Plant Research 129: 339–351. 27000875 10.1007/s10265-016-0807-2 PMC4850179

[CIT0020] Evers JB, Vos J, Chelle M, Andrieu B, Fournier C, Struik PC 2007 Simulating the effects of localized red:far-red ratio on tillering in spring wheat (*Triticum aestivum*) using a three-dimensional virtual plant model. New Phytologist 176: 325–336. 17888114 10.1111/j.1469-8137.2007.02168.x

[CIT0021] Evers J, Vos J, Yin X, Romero P, Van Der Putten P, Struik P 2010 Simulation of wheat growth and development based on organ-level photosynthesis and assimilate allocation. Journal of Experimental Botany 61: 2203–2216. 20231326 10.1093/jxb/erq025

[CIT0022] Evers JB, van der Krol AR, Vos J, Struik PC 2011 Understanding shoot branching by modelling form and function. Trends in Plant Science 16: 464–467. 21658989 10.1016/j.tplants.2011.05.004

[CIT0023] Feeny P 1976 Plant apparency and chemical defense. In: Wallace JW, Mansell RL, eds. Biochemical interaction between plants and insects. Boston, MA: Springer, 1–40.

[CIT0024] Fraser DP, Hayes S, Franklin KA 2016 Photoreceptor crosstalk in shade avoidance. Current Opinion in Plant Biology 33: 1–7. 27060719 10.1016/j.pbi.2016.03.008

[CIT0025] Giron D, Frago E, Glevarec G, Pieterse CMJ, Dicke M 2013 Cytokinins as key regulators in plant–microbe–insect interactions: connecting plant growth and defence. Functional Ecology 27: 599–609.

[CIT0026] Haag JJ, Coupe MD, Cahill JF 2004 Antagonistic interactions between competition and insect herbivory on plant growth. Journal of Ecology 92: 156–167.

[CIT0027] Hambäck PA, Beckerman AP 2003 Herbivory and plant resource competition: a review of two interacting interactions. Oikos 101: 26–37.

[CIT0028] Hättenschwiler S, Vitousek PM 2000 The role of polyphenols in terrestrial ecosystem nutrient cycling. Trends in Ecology and Evolution 15: 238–243. 10802549 10.1016/s0169-5347(00)01861-9

[CIT0029] Hemmerling R, Kniemeyer O, Lanwert D, Kurth W, Buck-Sorlin G 2008 The rule-based language XL and the modelling environment GroIMP illustrated with simulated tree competition. Functional Plant Biology 35: 739–750. 10.1071/FP08052 32688828

[CIT0030] Heuvelink E 1996 Dry matter partitioning in tomato: validation of a dynamic simulation model. Annals of Botany 77: 71–80.

[CIT0031] Hikosaka K, Anten NPR, Borjigidai A et al 2016 A meta-analysis of leaf nitrogen distribution within plant canopies. Annals of Botany 118: 239–247. 27296134 10.1093/aob/mcw099 PMC4970363

[CIT0032] Hirose T 2005 Development of the Monsi–Saeki theory on canopy structure and function. Annals of Botany 95: 483–494. 15585544 10.1093/aob/mci047 PMC4246794

[CIT0033] Hirose T, Werger MJA, Pons TL, Rheenen JWA 1987 Canopy structure and leaf nitrogen distribution in a stand of *Lysimachia vulgaris* L. as influenced by stand density. Oecologia 77: 145–150. 10.1007/BF00379180 28310366

[CIT0034] Izaguirre M, Mazza C, Astigueta M, Ciarla A, Ballaré C 2013 No time for candy: passionfruit (*Passiflora edulis*) plants down-regulate damage-induced extra floral nectar production in response to light signals of competition. Oecologia 173: 213–221. 23839264 10.1007/s00442-013-2721-9

[CIT0035] Jacquemoud S, Baret F 1990 PROSPECT: a model of leaf optical properties spectra. Remote Sensing of Environment 34: 75–91.

[CIT0036] Jing J, Raaijmakers C, Kostenko O, Kos M, Mulder PPJ, Bezemer TM 2015 Interactive effects of above- and belowground herbivory and plant competition on plant growth and defence. Basic and Applied Ecology 16: 500–509.

[CIT0037] Kang M, Heuvelink E, Carvalho SMP, de Reffye P 2012 A virtual plant that responds to the environment like a real one: the case for chrysanthemum. New Phytologist 195: 384–395. 22621431 10.1111/j.1469-8137.2012.04177.x

[CIT0038] Kim TN, Underwood N, Inouye BD 2013 Insect herbivores change the outcome of plant competition through both inter- and intraspecific processes. Ecology 94: 1753–1763. 24015519 10.1890/12-1261.1

[CIT0039] Koricheva J, Barton KE 2012 Temporal changes in plant secondary metabolite production: patterns, causes and consequences. In: Iason GR, Dicke M, Hartley SE, eds. The ecology of plant secondary metabolites: from genes to global processes. Cambridge: Cambridge University Press, 34–55.

[CIT0040] Lopez G, Favreau RR, Smith C, DeJong TM 2010 L-PEACH: a computer-based model to understand how peach trees grow. HortTechnology 20: 983–990.

[CIT0041] McKey D 1974 Adaptive patterns in alkaloid physiology. American Naturalist 108: 305–320.

[CIT0042] Moreno JE, Tao Y, Chory J, Ballare CL 2009 Ecological modulation of plant defense via phytochrome control of jasmonate sensitivity. Proceedings of the National Academy of Sciences, USA 106: 4935–40. 10.1073/pnas.0900701106 PMC2660767 19251652

[CIT0043] Niinemets Ü, Anten NPR 2009 Packing the photosynthetic machinery: from leaf to canopy. In: Laisk A, Nedbal L, Govindjee, eds. Photosynthesis in silico: understanding complexity from molecules to ecosystems. Dordrecht: Springer Netherlands, 363–399.

[CIT0044] Pantazopoulou CK, Bongers FJ, Küpers JJ et al 2017 Neighbor detection at the leaf tip adaptively regulates upward leaf movement through spatial auxin dynamics. Proceedings of the National Academy of Sciences, USA 114: 7450–7455. 10.1073/pnas.1702275114 PMC5514729 28652357

[CIT0045] Poelman EH, Kessler A 2016 Keystone herbivores and the evolution of plant defenses. Trends in Plant Science 21: 477–485. 26832946 10.1016/j.tplants.2016.01.007

[CIT0046] Poelman EH, Broekgaarden C, Van Loon JJA, Dicke M 2008 Early season herbivor differentially affects plant defence responses to subsequently colonizing herbivores and their abundance in the field. Molecular Ecology 17: 3352–3365. 18565114 10.1111/j.1365-294X.2008.03838.x

[CIT0047] Poelman EH, van Dam NM, van Loon JJA, Vet LEM, Dicke M 2009 Chemical diversity in *Brassica oleracea* affects biodiversity of insect herbivores. Ecology 90: 1863–1877. 19694135 10.1890/08-0977.1

[CIT0048] Rees M, Brown VK 1992 Interactions between invertebrate herbivores and plant competition. Journal of Ecology 80: 353–360.

[CIT0049] Schadler M, Brandl R, Haase J 2007 Antagonistic interactions between plant competition and insect herbivory. Ecology 88: 1490–1498. 17601141 10.1890/06-0647

[CIT0050] Stam JM, Kroes A, Li Y et al 2014 Plant interactions with multiple insect herbivores: from community to genes. Annual Review of Plant Biology 65: 689–713. 10.1146/annurev-arplant-050213-035937 24313843

[CIT0051] Stockhoff BA 1994 Maximization of daily canopy photosynthesis – effects of herbivory on optimal nitrogen distribution. Journal of Theoretical Biology 169: 209–220.

[CIT0052] Vos J, Evers JB, Buck-Sorlin GH, Andrieu B, Chelle M, de Visser PHB 2010 Functional–structural plant modelling: a new versatile tool in crop science. Journal of Experimental Botany 61: 2101–2115. 19995824 10.1093/jxb/erp345

[CIT0053] de Vries J, Evers JB, Poelman EH 2017 Dynamic plant–plant–herbivore interactions govern plant growth–defence integration. Trends in Plant Science 22: 329–337. 28089490 10.1016/j.tplants.2016.12.006

[CIT0054] de Wit M, Spoel SH, Sanchez-Perez GF et al 2013 Perception of low red:far-red ratio compromises both salicylic acid- and jasmonic acid-dependent pathogen defences in Arabidopsis. The Plant Journal 75: 90–103. 23578319 10.1111/tpj.12203

[CIT0055] Yin X, Goudriaan JAN, Lantinga EA, Vos JAN, Spiertz HJ 2003 A flexible sigmoid function of determinate growth. Annals of Botany 91: 361–371. 12547689 10.1093/aob/mcg029 PMC4244967

[CIT0056] Zhu J, van der Werf W, Anten NPR, Vos J, Evers JBC 2015 The contribution of phenotypic plasticity to complementary light capture in plant mixtures. New Phytologist 207: 1213–1222. 25898768 10.1111/nph.13416

[CIT0057] Zust T, Agrawal AA 2017 Trade-offs between plant growth and defense against insect herbivory: an emerging mechanistic synthesis. Annual Review of Plant Biology 68: 513–534. 10.1146/annurev-arplant-042916-040856 28142282

