# Supplementary material for: Elucidating the interaction between light competition and herbivore feeding patterns using functional–structural plant modelling
Source: Ann Bot. 2018 Jan 24;121(5):1019–31. doi: 10.1093/aob/mcx212 (PMC5906910; doi:10.1093/aob/mcx212)
Supplement: mcx212_suppl_Supplementary_Information [file mcx212_suppl_supplementary_information.docx]

## Supporting Information

Article title: Elucidating the interaction between light competition and herbivore feeding patterns using functional-structural plant modelling

Authors: Jorad de Vries, Erik H. Poelman, Niels Anten and Jochem B. Evers

The following Supporting Data is available for this article:

**Methods S1; Experimental supplementary**

*Leaf angles –* leaf angles were measured using ImageJ from pictures taken in the field in July 2014.

*Branching angles -* branching angles were measured using ImageJ from pictures taken in the field in July 2014.

*Ear development –* on the 14^th^ and 21^st^ of July 2014, we counted the number of pods on the main stem ear of the 9 selected plants in the three densities. The plants made on average 23.8±0.2 pods (no difference between densities) over this 7 day period, which spanned 160.5 growing degree days (gdd) and translates to 6.74 gdd per pod. We counted the number of flowers per ear, which averaged at 11.4±0.15 flowers per ear and translates to a flower life time of 77 gdd (11.4 flowers * 6.74 gdd per pod).

*Photosynthetic capacity –* in July 2014 and 2015 we selected one plant in the low density and used a LICOR (LI-6400XT, LI-COR Biosciences, Lincoln, Nebraska,USA) to measure the photosynthetic capacity of 9 mature leaves along the main stem. The photosynthetic capacity ranged from 32 µmol m^-2^ s^-1^ to 2.1 µmol m^-2^ s^-1^, which led us to use a base A_max_ of 30 µmol m^-2^ s^-1^ for out model.

*Light quality (RFR) –* on the 1^st^ of July 2014 we measured the R:FR ratio in the three densities using a Skye SKR100/116 Fibre Optic Probe Measuring System (Skye Instruments Ltd, Powys, UK). We randomly selected 5 plants per density and measured the R:FR in four directions at 5 and 20 cm from the ground while pointing the sensor away from the selected plant.

**Methods S2; Model design supplementary**

*Assimilate production and organ growth*

A leaf can adjust its maximum rate of photosynthesis (*A_max_*) based on the relative light interception (*relPAR, dimentionless*) and the maximum rate of photosynthesis of light grown leaves (*A_max0_*).

$$A_{max}=\left( A_{max0} \right)^{relPAR*0.4}$$

Gross photosynthesis rate (*A_G_, µmol CO_2_ m^-2^ s^-1^*) is calculated using the absorbed photosynthetically active radiation (*APAR, µmol*), the initial slope of the light response curve (*ε, µmol CO_2_ µmol^-1^*) and the maximum rate of photosynthesis (*A_max_ g CO_2_ m^-2^ d^-1^)*.

$$A_{G}=A_{max}*(1-\exp\left( -\varepsilon*\frac{APAR}{A_{max}} \right))$$

Net photosynthesis (*A_N_, µmol CO_2_ m^-2^ s^1^)* accounts for dark respiration (*R_d_, µmol CO_2_)*.

$$A_{N}=A_{G}-R_{d}$$

The assimilated CO_2_ is converted to produce sugars, a conversion parameter (*C*) converts from µmol CO_2_ to grams of glucose and seconds to hours, which is then multiplied by the daylength (*hours*).

$$\frac{dSugar}{dt}=C*daylength*A_{N}$$

The assimilated carbon is distributed over the plant architecture to facilitate plant growth. The model distributes assimilates over the plant architecture by using a centralised carbon pool (*S, gram sugar*). Each growing organ will have a demand for assimilates expressed as a sink strength (*Sink, g sugar*), that includes assimilates needed to achieve potential growth (*Growth_pot_, g sugar*) as well as costs associated with construction of biomass (*cc, , dimentionless > 1*) and respiration (*rm, dimentionless*):

$$Sink= Growth_{pot}*cc+biomass*rm$$

Potential growth is calculated differently for different types of organs, depending on their geometrical and biological properties. The change in leaf length (*dL/dt, m)* is calculated using the Yin functions for sigmoid growth (Yin 2003), where t_e_ represents the growth duration (*gdd*), t_m_ the point at which growth is maximal (*gdd*), cm is the growth at age t_m_. w_max_ is the potential size of the organ (*m*) and t is the organ age (*gdd*).

$$\frac{dL}{dt}=c_{m}\left( \left( \frac{t_{e}-t}{t_{e}-t_{m}} \right)\left( \frac{t}{t_{m}} \right)^{\frac{t_{m}}{t_{e}-t_{m}}} \right)$$

$$cm=w_{max}\left( \left( \frac{{2*t}_{e}-t_{m}}{t_{e}\left( t_{e}-t_{m} \right)} \right)\left( \frac{t_{m}}{t_{e}} \right)^{\frac{t_{m}}{t_{e}-t_{m}}} \right)$$

The value of *w_max_* is based on empirical data of leaf length-rank profiles (*w_max0_, m*) and density effects. The lowest of the local or systemic R:FR signal (*rfr*) and a number of parameters such as a leaf growth inhibition parameter (*LLrfr = 0.4*) calculate the eventual value of *w_max_*.

$$w_{max}=w_{max0}*\left( 1-\frac{1-LLrfr}{1+\exp\left( s*\left( rfr-m \right) \right)} \right)$$

Potential growth is calculated using a width:length ratio parameter (*r_wl_*) based on the leafs R:FR signal, the current and potential new leaf length (*L_0_ and L_1_, m*), the current and potential new leaf width (*W_0_ and W_1_, m*), a leaf shape conversion factor (*L_area_*) and the leaf mass per unit area (*LMA, g m^-2^*).

$$L_{1}=L_{0}+\frac{dL}{dt}$$

$$W_{1}=max(W_{0}, r_{wl}*L_{1})$$

$$Growth_{pot}=(L_{1}*W_{1}-L_{0}*W_{0})*L_{area}*LMA$$

$$r_{wl}=WL_{max}-\frac{WL_{max}-WL_{min}}{1+\exp\left( s*\left( rfr-m \right) \right)}$$

Carbon allocation (*CA, g*) is proportional to the sum of sinks (*ss, g sugar*). This ensures that in carbon limiting conditions all growing organs have their growth reduced with an equal percentage.

$$CA= min\left( Sink, S*\frac{Sink}{SS} \right)$$

The amount of carbon available for growth (*CFlux, g sugar*) then has to account for construction costs and maintenance respiration.

$$CFlux=\frac{CA-biomass*rm}{cc}$$

The carbon allocated to the organ leads to a change in biomass, which will translate in a change in organ size.

$$\frac{dbiomass}{dt}=CFlux$$

$$Area=biomass/LMA$$

$$W_{new}=\sqrt{r_{wl}*\frac{Area}{L_{area}}}$$

$$L_{new}=\frac{W}{r_{wl}}$$

However, since the width:length ratio is variable depending on light conditions we must account for the fact that leaves cannot become shorter or narrower.

$W= \frac{Area}{L_{area}}*\frac{1}{L}$ $if L_{new}<L$

$L= \frac{Area}{L_{area}}*\frac{1}{W}$ $if W_{new}<W$

Internode growth is described using two independent mechanisms; internode length growth, which is affected by local rfr conditions, and internode radial growth, which is described using a pipe model. Internode length is described using the Yin function, with potential length (*wmax, g*) being calculated using the potential size in low densities (*w_max0_, g*) multiplied by an elongation parameter (*Elong, dimentionless > 1*) dependent on local rfr signalling.

$wmax=w_{max0}*Elong$ if $rfr<threshold$

The pipe model assumes that the cross-sectional surface area of an internode equals the sum of the cross-sectional surface area of its daughter internodes. The new radius of and internode (*r_1_, m*) thus depends on the radii of its daughter internodes (*r_a_ and r_b_, m*) as follows;

$$r_{1}=\sqrt{\frac{\pi r_{a}^{2}+\pi r_{b}^{2}}{\pi}}$$

The potential growth of the internode is the difference between current and new volume multiplied by the internode tissue density (*TD_I_, g m^-3^*).

$$Growth_{pot}=\left( L_{1}*2*\pi*r_{1}-L_{0}*2*\pi*r_{0} \right)*TD_{I}$$

The potential growth is translated to a sink strength and carbon flux in the same way as happens with the leaves. The allocated assimilates then lead to a change in internode size proportional to the ratio between the assimilates available for growth and the demand for those assimilates.

$$\frac{dlength}{dt}=(L_{1}-L_{0})*\frac{CA-biomass*rm}{Sink-biomass*rm}$$

$$\frac{dradius}{dt}={(r}_{1}-r_{0})*\frac{CA-biomass*rm}{Sink-biomass*rm}$$

*Branching*

Axillary meristems can grow and develop similar to the apical shoot meristem to form a branch. Branch initiation and abortion is simulated using cues that correspond to apical dominance, assimilate availability and light quality. Apical dominance is represented as a period of time after initiation of an axillary meristem during which it is not allowed to grow out into a branch (*age>MeristemDev*), or when the dominance of the apical meristem is lifted because it becomes generative. Additionally, a bud needs enough sugar (*SugarStatus>SugarThreshold*) to break and develop into a branch. Detection of neighbouring plants through an R:FR signal (see shade avoidance) or senescence or removal of the maternal leaf can abort a branch between the stage where it has formed three leaves (personal observation) up to when the axillary meristem becomes generative.

*Ear development*The potential growth of an ear is determined by the flower appearance rate (6.7 gdd per flower), the time it takes a flower to mature into a pod (77 gdd), as well as the potential seed mass of a pod. An ear can make a maximum of 80 pods, which contain 8 seeds with a potential weight of 8.37·10^-5^ g per seed.


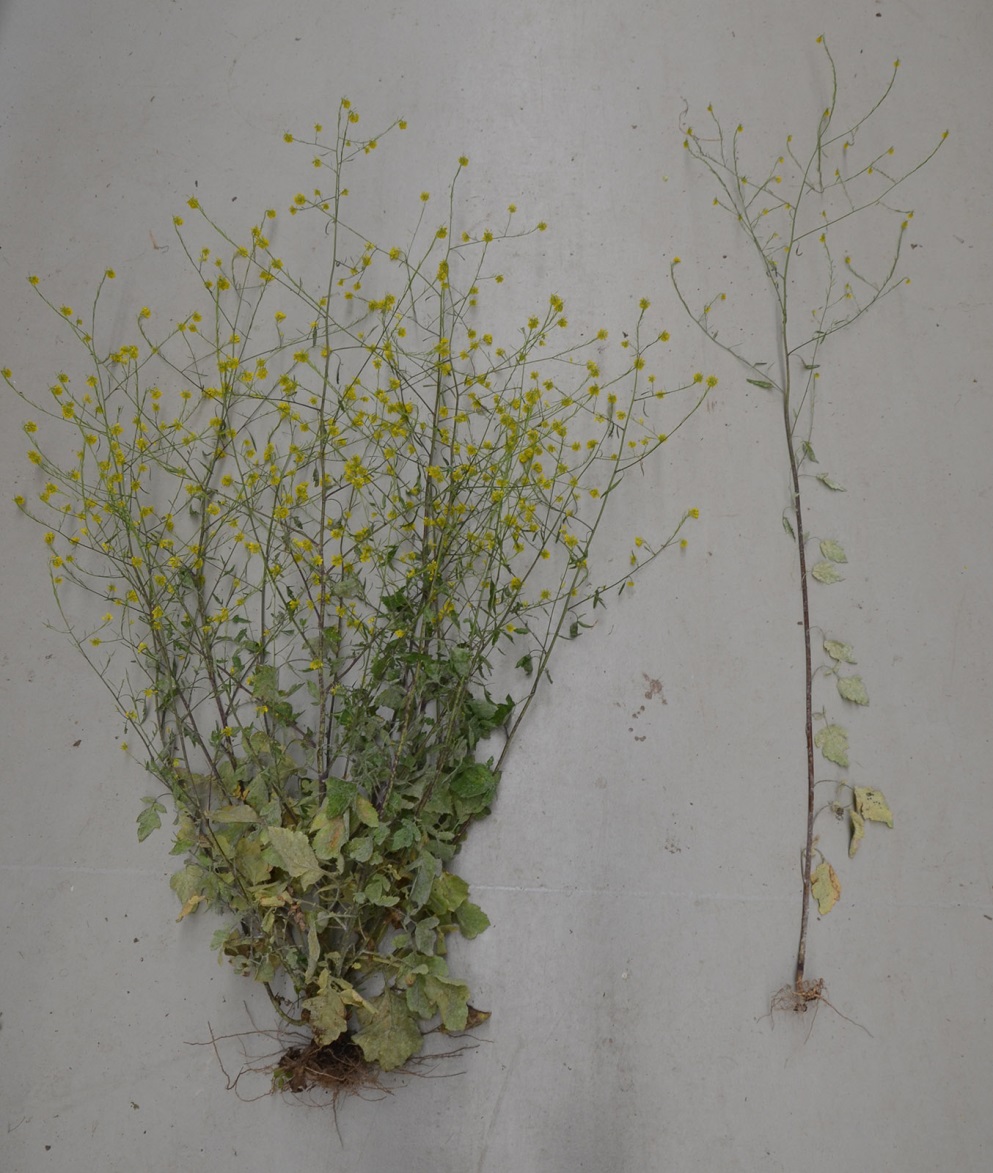


**Figure S1.** Field plants grown in a low density of 1 plant/m^2^ (left) and high density of 25 plants/m^2^ (right) after 124 days of growth.


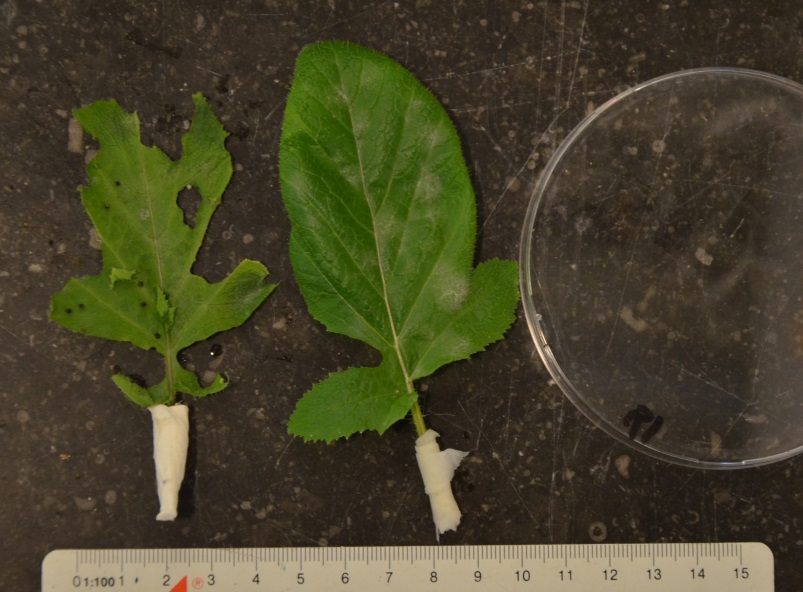


**Figure S2.** Two leaves used in the caterpillar greenhouse experiment, one after two days of caterpillar feeding (left) and one fresh leaf (right).


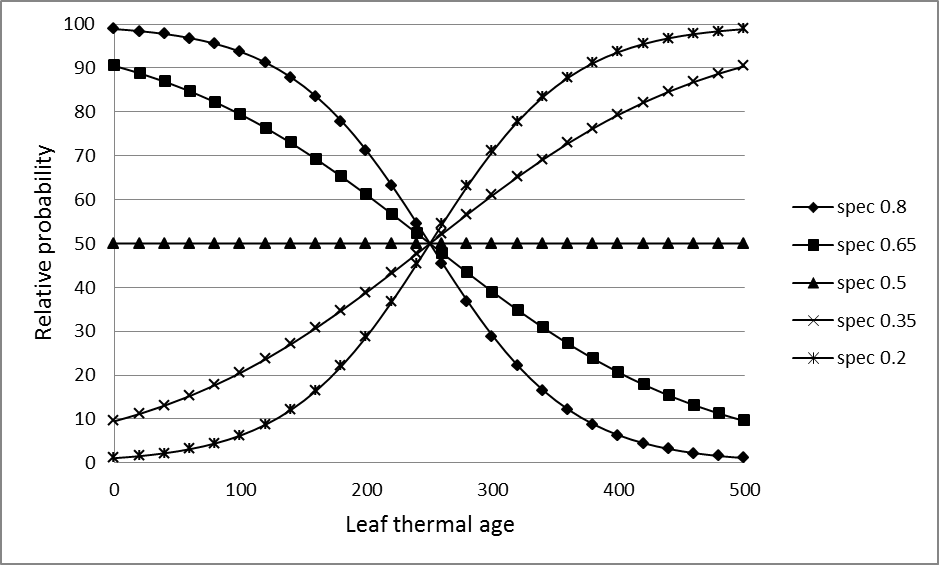


**Figure S3.** The relative probability a leaf is selected for feeding by a herbivore of a given level of specialisation (*h*) as a function of the leafs age in degree days. A high *h* parameter denotes a high level of herbivore specialisation and a high preference for young leaves, while a low spec parameter denotes a generalist herbivore with a feeding preference for older leaves.


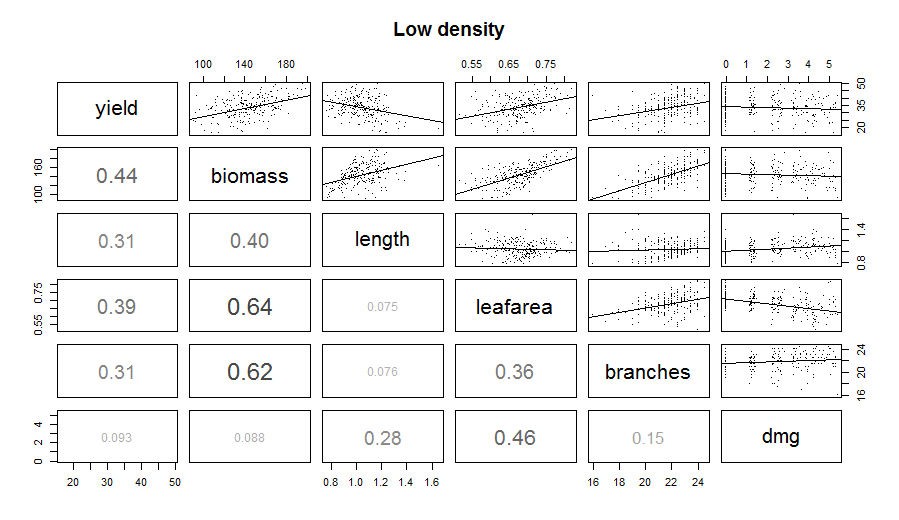

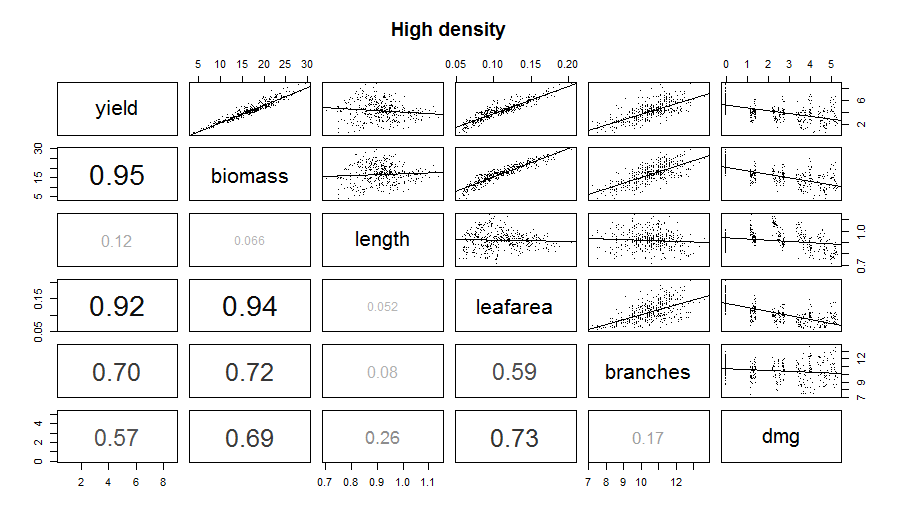

**Figure S4.** Correlations between six plant variables of an increasing level of integration within the modelling structure: herbivore damage (dmg), the number of branches (branches), max leaf area (leafarea), plant height (length), plant biomass (biomass), plant yield (yield). Regression lines are drawn through the point clouds plotted in the top panels. Corresponding R^2^ values are plotted in the bottom panels and show how much of the variation in the variable of a higher level of integration is explained by the variable of the lower level of integration.


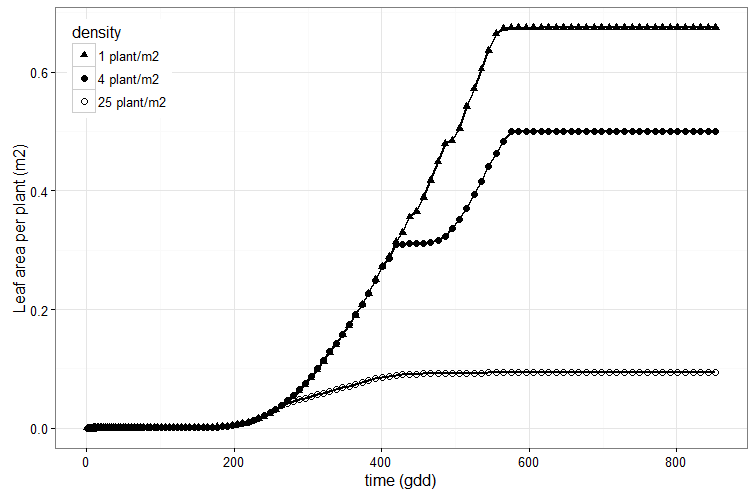


**Figure S5.** Simulated leaf area per plant (m^2^) over time (gdd) in three densities (1,4 and 25 plants/m^2^).


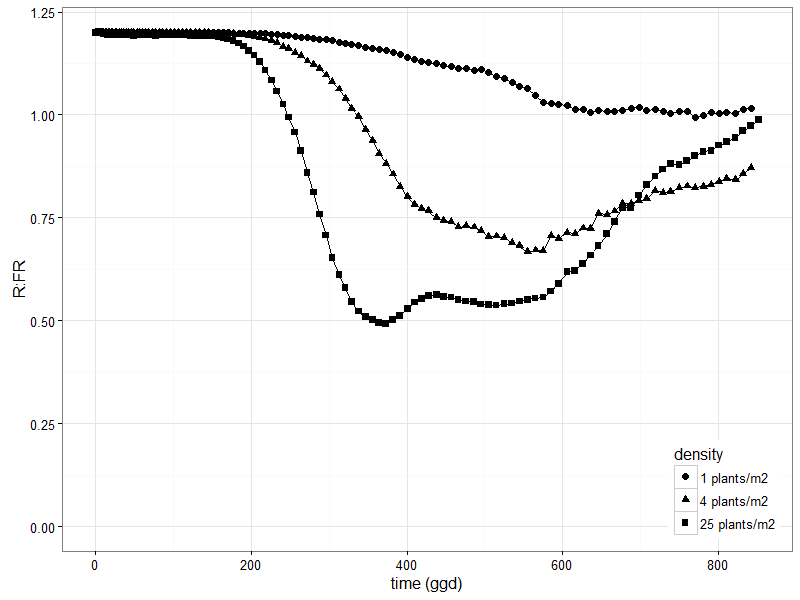


**Figure S6.** Simulated R:FR ratio over time (gdd) in three densities (1,4 and 25 plants/m2).


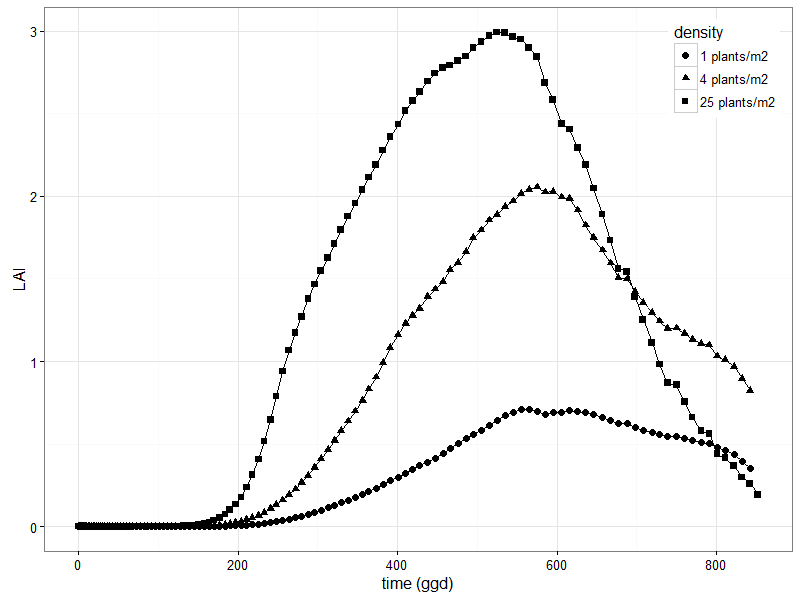


**Figure S7.** Simulated leaf area index (LAI) over time (gdd) in three densities (1,4 and 25 plants/m^2^).
